# *HWA1*- and *HWA2*-Mediated Hybrid Weakness in Rice Involves Cell Death, Reactive Oxygen Species Accumulation, and Disease Resistance-Related Gene Upregulation

**DOI:** 10.3390/plants8110450

**Published:** 2019-10-25

**Authors:** Kumpei Shiragaki, Takahiro Iizuka, Katsuyuki Ichitani, Tsutomu Kuboyama, Toshinobu Morikawa, Masayuki Oda, Takahiro Tezuka

**Affiliations:** 1 Graduate School of Life and Environmental Sciences, Osaka Prefecture University, 1-1 Gakuen-cho, Nakaku, Sakai, Osaka 599-8531, Japan; ma201027@edu.osakafu-u.ac.jp (K.S.); ddlfte@gmail.com (T.I.); d-morikawa@hannan-u.ac.jp (T.M.); masa-oda@d.email.ne.jp (M.O.); 2 Faculty of Agriculture, Kagoshima University, 1-21-24 Korimoto, Kagoshima, Kagoshima 890-0065, Japan; ichitani@agri.kagoshima-u.ac.jp; 3 College of Agriculture, Ibaraki University, 3-21-1 Chuo, Ami, Ibaraki 300-0393, Japan; tsutomu.kuboyama.a@vc.ibaraki.ac.jp; 4 Education and Research Field, College of Life, Environment, and Advanced Sciences, Osaka Prefecture University, 1-1 Gakuen-cho, Nakaku, Sakai, Osaka 599-8531, Japan

**Keywords:** *Oryza sativa*, hybrid weakness, cell death, reactive oxygen species, leaf yellowing, SPAD, hypersensitive response

## Abstract

Hybrid weakness is a type of reproductive isolation in which F_1_ hybrids of normal parents exhibit weaker growth characteristics than their parents. F_1_ hybrid of the *Oryza sativa* Indian cultivars ‘P.T.B.7′ and ‘A.D.T.14′ exhibits hybrid weakness that is associated with the *HWA1* and *HWA2* loci. Accordingly, the aim of the present study was to analyze the hybrid weakness phenotype of the ‘P.T.B.7′ × ‘A.D.T.14′ hybrids. The height and tiller number of the F_1_ hybrid were lower than those of either parent, and F_1_ hybrid also exhibited leaf yellowing that was not observed in either parent. In addition, the present study demonstrates that SPAD values, an index correlated with chlorophyll content, are effective for evaluating the progression of hybrid weakness that is associated with the *HWA1* and *HWA2* loci because it accurately reflects degree of leaf yellowing. Both cell death and H_2_O_2_, a reactive oxygen species, were detected in the yellowing leaves of the F_1_ hybrid. Furthermore, disease resistance-related genes were upregulated in the yellowing leaves of the F_1_ hybrids, whereas photosynthesis-related genes tended to be downregulated. These results suggest that the hybrid weakness associated with the *HWA1* and *HWA2* loci involves hypersensitive response-like mechanisms.

## 1. Introduction

The traits of existing crop cultivars can be improved by crossing cultivars or lines to introduce beneficial traits, such as resistance or tolerance to disease or stress, to susceptible cultivars. However, because reproductive isolation mechanisms can hinder the production of hybrids, methods must be developed to overcome the underlying mechanisms of such reproductive isolation.

One type of post-zygotic reproductive isolation, namely hybrid weakness (i.e., hybrid lethality or hybrid necrosis). F_1_ hybrids that exhibit this phenomenon are characterized by weaker growth than their parents, and the phenomenon has been reported to occur in the offspring of crosses involved a number of species, including *Oryza sativa* [1,2,3,4], *Nicotiana* spp. [5], *Capsicum* spp. [6], *Arabidopsis thaliana* [7], *Triticum* spp. [8,9], *Gossypium* spp. [10], and *Phaseolus vulgaris* [11]. The genetic mechanisms of hybrid weakness are explained by the Bateson–Dobzhanzky–Muller model [12,13,14], which posits that the reduced hybrid vigor is driven by deleterious interactions between genes at different loci. In many cases, one of the causal genes is related to disease resistance (*R*), and interactions between the *R* gene and other causal gene cause autoimmune responses in the hybrid offspring [2,7]. The autoimmune responses include the accumulation of reactive oxygen species such as H_2_O_2_, cell death, upregulation of disease resistance-related genes, and downregulation of photosynthesis-related genes [2,7,15,16].

In rice, hybrid weakness has been reported to result from interactions between the *HWI1* locus, which encodes the LRR-RLK gene (*R* gene), and the *HWI2* locus, which encodes a subtilisin-like protease, and hybrids have been reported to exhibit localized programmed cell death (PCD), the high accumulation of salicylic and jasmonic acids, and amplified heat-related weakness symptoms [2]. These results demonstrate that the interaction of causal genes can activate downstream immune responses, such as hypersensitive response-like mechanisms [2,7].

The hybrid weakness that results from the interaction of *Hwa1-1*, a dominant allele of the *HWA1* locus, and *Hwa2-1*, a dominant allele of the *HWA2* locus, was firstly reported in rice by Oka [4]. In that study, F_1_ hybrid seedlings that exhibited hybrid weakness were reported to exhibit normal germination and seedling growth until developing three to four leaves, after which plant growth halted and the leaves yellowed. Then, unless the environment was particularly favorable, the plants died before reaching anthesis. The distributions of the *Hwa1-1* and *Hwa2-1* alleles were limited to Indian cultivars [4], and both the *HWA1* and *HWA2* loci were located in a 1637-kb region of the long arm of chromosome 11 [17]. However, the causal genes have not been identified, and the molecular mechanism underlying the hybrid weakness associated with the *HWA1* and *HWA2* loci remain unclear.

Accordingly, the aim of the present study was to characterize the phenotypes of the hybrid weakness that is associated with the *HWA1* and *HWA2* loci, in order to understand the system’s underlying mechanisms. The effectiveness of SPAD values, an index correlated with chlorophyll content [18], for determining the progression of the hybrid weakness was also evaluated. The occurrence of cell death and H_2_O_2_ accumulation was also evaluated, and the expression of disease resistance and photosynthesis-related genes in the leaves of F_1_ hybrids exhibiting hybrid weakness were analyzed.

## 2. Results

### 2.1. Hybrid Weakness Phenotypes

**Figure 1:**
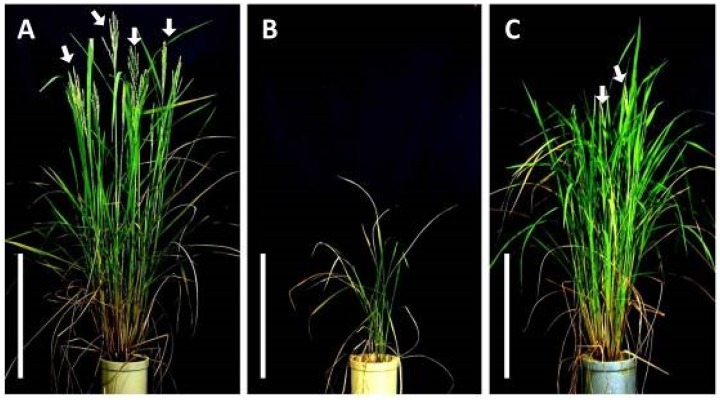
Parental and F_1_ hybrid phenotypes at 80 days after sowing. (**A**) *Oryza sativa* ‘A.D.T.14*′*; (**B**) F_1_ hybrid; and (**C**) *O. sativa* ‘P.T.B.7*′*. Arrows indicate emerging panicles. Scale bars indicate 50 cm.

**Figure 2:**
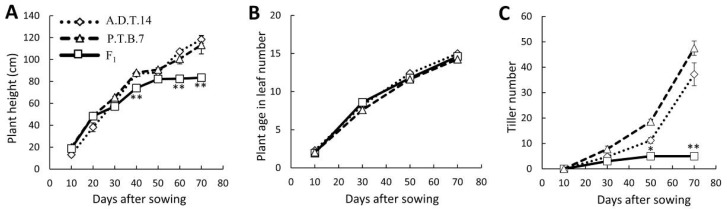
Phenotypic traits of parental and F_1_ hybrid rice. (**A**) plant height; (**B**) plant age in leaf number; and (**C**) tiller number. Values and error bars indicate mean ± SE values (*n* = 5), although some error bars are hidden by the symbols. Mid-parental and hybrids values were compared using two-tailed Student’s t-test. Significance: ** *P* < 0.01, * *P* < 0.05.

The Oryza sativa Indian cultivars ‘A.D.T.14*′* and ‘P.T.B.7*′* carry homozygous Hwa1-1 and Hwa2-1 alleles, respectively. All the F_1_ hybrids of a cross between ‘A.D.T.14*′* and ‘P.T.B.7*′* exhibited dwarfism, reduced tiller number, and leaf yellowing (Figure 1). Increases in the height of the F_1_ hybrids nearly halted at 50 days after sowing (DAS), whereas that of the parents continued increasing (Figure 2A). The progression of plant age in leaf number in the F_1_ hybrids was the same as that in both parents (Figure 2B). The tiller number of both parents continued increasing and reached >35 tillers at 70 DAS, whereas that of the F_1_ hybrids increased little and only reached five tillers by 70 DAS (Figure 2C). In addition, both parents headed by 80 DAS, whereas none of the F_1_ hybrids had started heading after 140 DAS (Figure 1).

**Figure 3:**
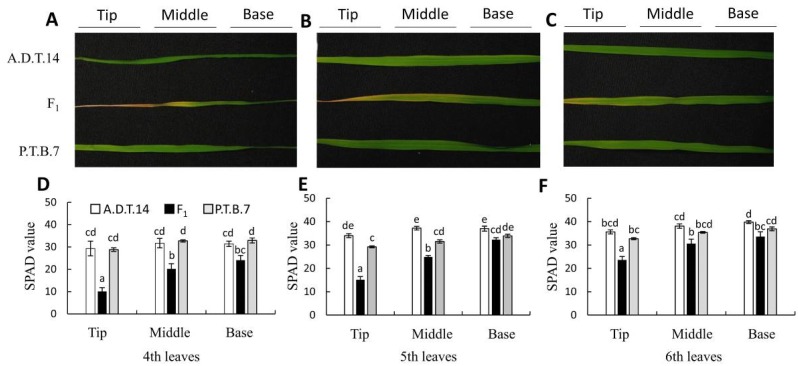
Phenotypes and SPAD values of leaves (fourth to sixth) from parental and F_1_ hybrid rice. The phenotypes (**A**–**C**) and SPAD values (**D**–**F**) of fourth (**A**, **D**), fifth (**B**, **E**), and sixth (**C**, **F**) leaves were assessed at 60 days after sowing. SPAD value was measured at the tip, middle, and base of each leaf. Values and error bars indicate mean ± SE values (*n* = 3). Different lowercase letters in each plot (**D**–**F**) indicate significant differences (Tukey HSD test, *P* < 0.05).

Leaf yellowing was first observed in the F_1_ hybrids that had developed seventh or eighth leaves at 30 DAS. Afterward, the leaves turned yellow sequentially, from the lower to the upper leaves. At 60 DAS, the fourth, fifth, and sixth leaves of the F_1_ hybrids turned yellow, starting from the leaf tip, and progressing toward the leaf base, whereas those of both parents remained green (Figure 3A–C). Meanwhile, the SPAD values of the fourth, fifth, and sixth leaves of the F_1_ hybrids were lower than those of the parents (Figure 3D–F). Furthermore, in the leaves of the F_1_ hybrids, the SPAD values of the lower leaves were lower than those of the upper leaves (fourth vs. fifth and sixth leaves and fifth vs. sixth leaves), and within each leaf, the SPAD values of the leaf tips were lower than those of the leaf bases (Figure 3D–F).

### 2.2. Cell Death and H_2_O_2_ Accumulation

**Figure 4:**
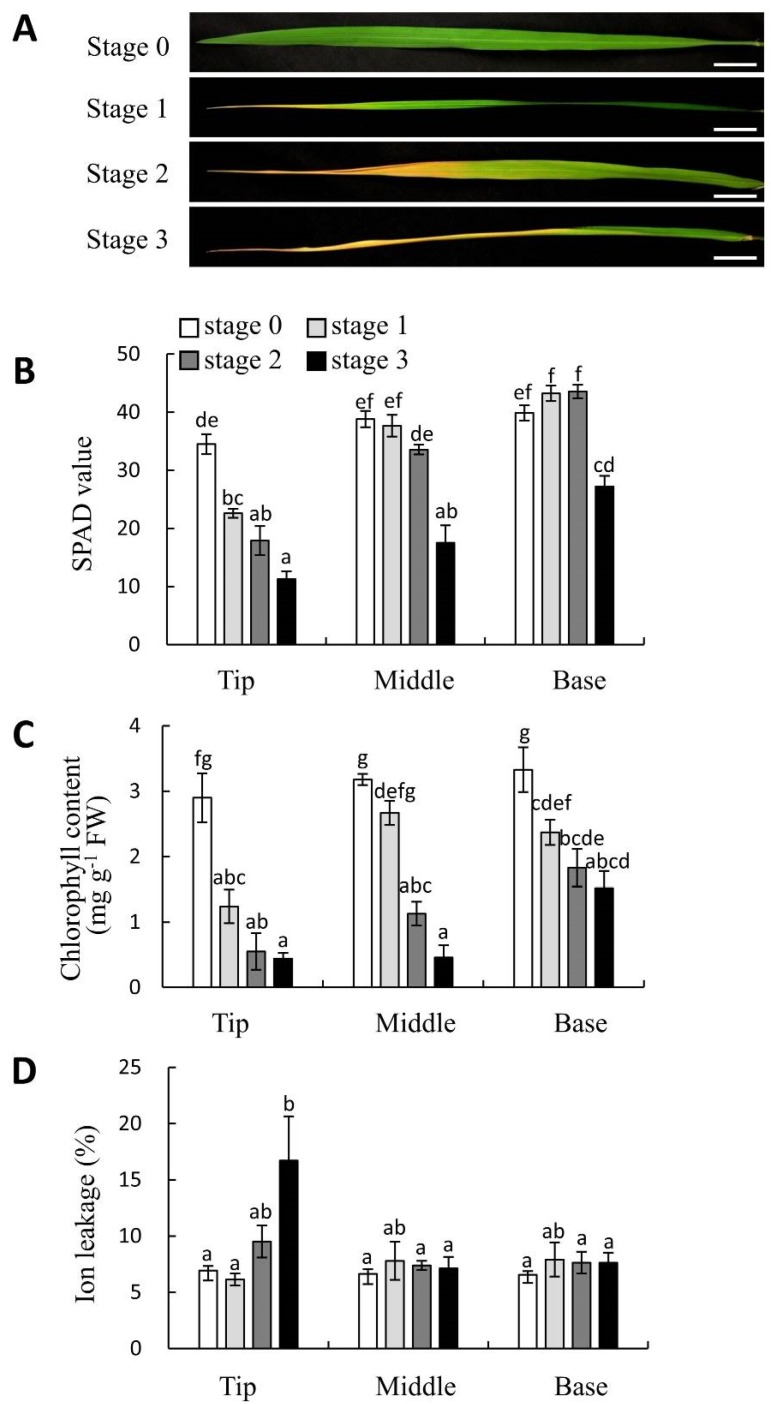
Physiological changes of yellowing hybrid leaves. (**A**) Stages of yellowing: Stage 0, no yellowing; Stage 1, 1/4 of leaf yellow; Stage 2, 1/2 of leaf yellow; Stage 3, 3/4 of leaf yellow. Scale bars indicate 2 cm. (**B**) Changes in the SPAD values at the tip, middle, and base of leaves during the progression of yellowing. (**C**) Changes in chlorophyll content during the progression of yellowing. (**D**) Changes in ion leakage during the progression of yellowing. Values and error bars (**B**–**D**) indicate mean ± SE values (*n* = 3), and different lowercase letters in each plot (**B**–**D**) indicate significant differences (Tukey HSD test, *P* < 0.05).

The physiological changes that accompanied leaf yellowing were surveyed by analyzing F_1_ hybrid leaves that had been classified into four stages based on degree of yellowing (Figure 4A). Chlorophyll content was assessed by SPAD analysis and spectrophotometry. The SPAD values of Stage-1, -2, and -3 leaf tips, Stage-3 leaf middles, and Stage-3 leaf bases were lower than those of Stage-0 leaves (Figure 4B). The SPAD values of Stage-3 leaf tips, Stage-3 leaf middles, and Stage-3 leaf bases were lower than those of Stage-1 leaves (Figure 4B). The SPAD values of Stage-3 leaf middles and Stage-3 leaf bases were lower than those of Stage-2 leaves (Figure 4B). Total chlorophyll content also decreased in all leaf parts (tip, middle, and base) as yellowing progressed (Figure 4C). Because of the usefulness of SPAD value as discussed later, the progression of yellowing of leaves used in the subsequent experiments were evaluated based on SPAD value.

**Figure 5:**
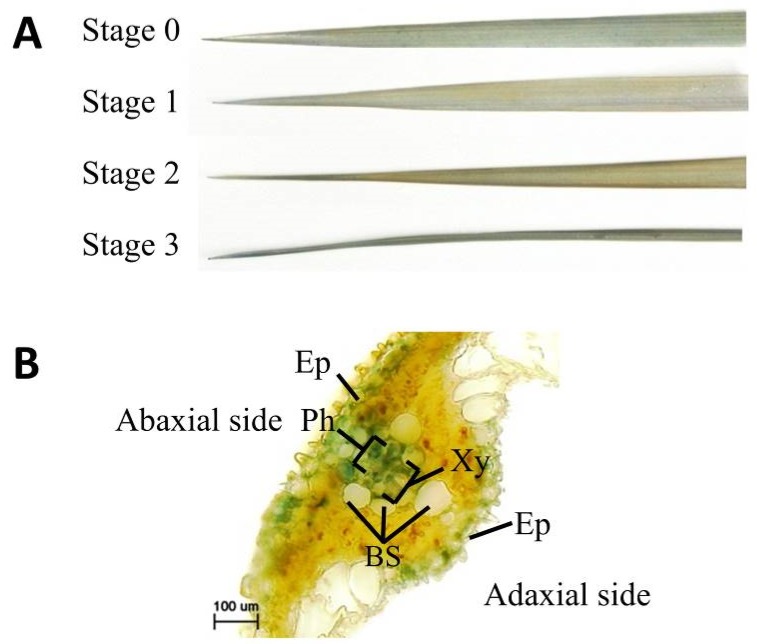
Trypan blue staining of dead cells in F_1_ leaves. (**A**) Stained leaves from each yellowing stage. (**B**) Transverse section of a stained Stage-3 leaf. Scale bar indicates 100 µm. Xy: Xylem; Ph: Phloem; Ep: Epidermal cell; BS: Bundle sheath cell.

To determine whether cell death occurred in F_1_ hybrid leaves, cellular ion leakage, owing to ion permeability by cell death, was measured. Ion leakage increased slightly and significantly in the tips of Stage-2 and Stage-3 leaves, respectively (Figure 4D). Cell death in F_1_ hybrid leaves was also evaluated using trypan blue staining, which is used to identify the highly permeable membranes of dead cells. Only Stage-3 leaves contained dead cells (Figure 5A), and the analysis of transverse sections of Stage 3 revealed that the dead cells were located around vascular and epidermal cells (Figure 5B).

**Figure 6:**
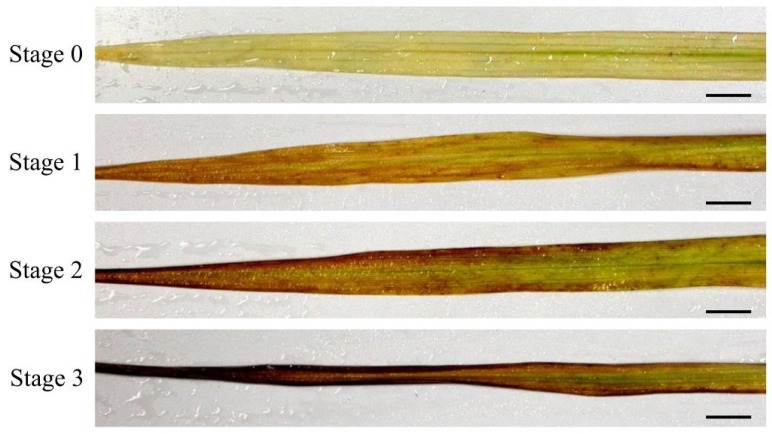
Presence of reactive oxygen species in hybrid leaves. 3,3-diaminobenzidine (DAB) staining was used to detect H_2_O_2_. Scale bars indicate 1 cm.

Meanwhile, 3,3-diaminobenzidine (DAB) staining revealed the presence of H_2_O_2_, which, as a reactive oxygen species, is an important regulator of cell death. Plant tissue is stained brown when DAB is oxidized by H_2_O_2_ into an insoluble polymer. Hydrogen peroxide (H_2_O_2_) was detected in the leaves of all stages, except Stage 0 (Figure 6).

### 2.3. Hybrid Weakness-Related Gene Expression

**Figure 7:**
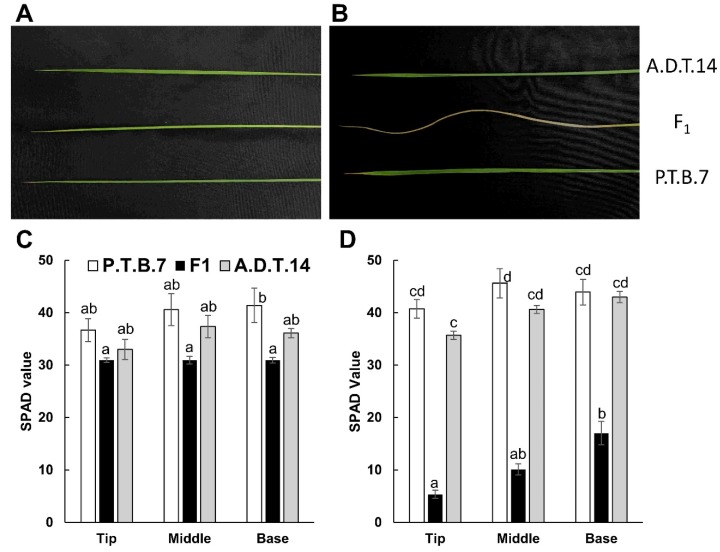
Phenotypes and SPAD values of the parental and F_1_ leaves (11th and 13th) used for gene expression analysis. The phenotypes (**A**, **B**) and SPAD values (**C**, **D**) of 11th (**A**, **C**) and 13th (**B**, **D**) leaves were assessed at 70 d after sowing. SPAD value was measured at the tip, middle, and base of each leaf. Values and error bars indicate mean ± SE values (*n* = 3), and different lowercase letters in each plot (**C**, **D**) indicate significant differences (Tukey HSD test, *P* < 0.05).

At 70 DAS, the 11th (Stage 3) and 13th (Stage 0) leaves of the parents and F_1_ offspring were collected for gene expression analysis (Figure 7). The 13th leaves of both the parents and hybrids were entirely green, as indicated by high SPAD values, even though the SPAD values of the F_1_ hybrids were somewhat lower than those of either parent (Figure 7A,C). Meanwhile, the 11th leaves of the F_1_ hybrids exhibited significant yellowing, as indicated by low SPAD values, whereas those of both parents were entirely green, as indicated by high SPAD values (Figure 7B,D).

**Figure 8:**
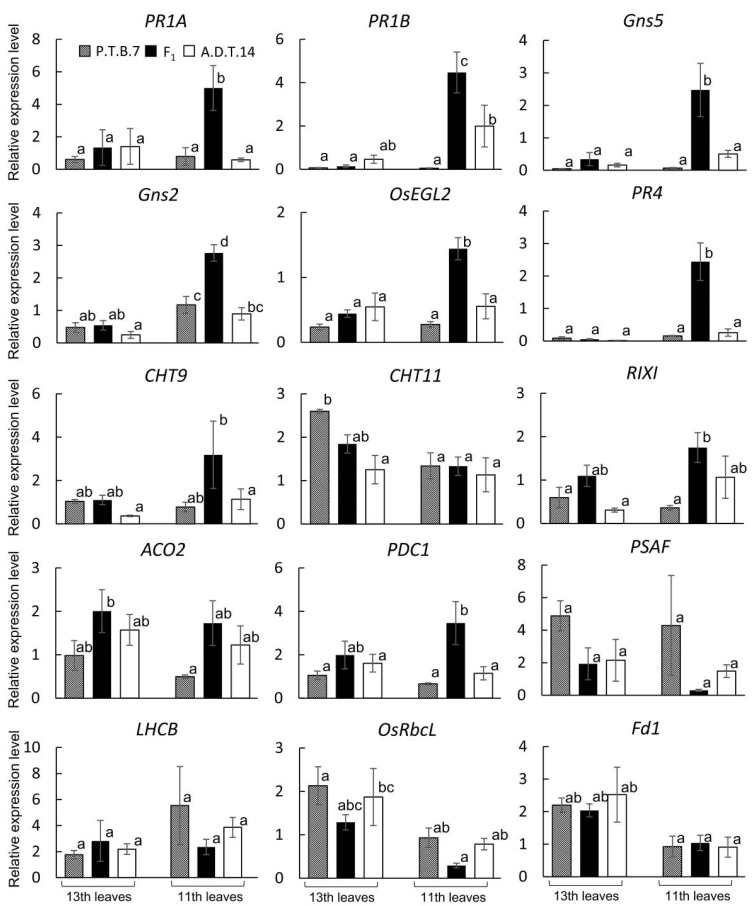
Relative gene expression levels of parental and F_1_ hybrid leaves between ‘P.T.B.7′ and ‘A.D.T.14′. Values and error bars indicate mean ± SE values (*n* = 3), and different lowercase letters in each plot indicate significant differences (Tukey HSD test, *P* < 0.05).

The expression of 11 disease resistance-related genes and four photosynthesis-related genes were surveyed (Table 1 and Table S1). The PR1 genes (PR1A and PR1B), the expression of which is induced by salicylic acid [19,20], were upregulated in the 11th leaves of the F_1_ hybrids (Figure 8), as were several PR2 genes (Gns5, Gns2, and OsEGL2), which encode glucanase-related proteins that degrade fungal cell walls [21] (Figure 8). Meanwhile, of several genes that encode chitinase-related proteins (PR4, CHT9, CHT11, and RIXI), which also degrade fungal cell walls [22], PR4, CHT9, and RIXI were all upregulated in the 11th leaves of the F_1_ hybrids; however, only PR4 was upregulated significantly (Figure 8). The expression of ACO2, which encodes an enzyme related to ethylene production [23], was similar in the 11th and 13th leaves of the F_1_ hybrids (Figure 8). Finally, PDC1, the expression of which is induced by jasmonic acid [24], was upregulated in the 11th leaves of the F_1_ hybrids, although not significantly (Figure 8).

Of the four photosynthesis-related genes, PSAF, LHCB, and OsRbcL were somewhat downregulated in the 11th leaves of the F_1_ hybrids; however, none of these trends were significant (Figure 8).

## 3. Discussion

Oka [4] reported that F_1_ hybrids that exhibit hybrid weakness associated with the *HWA1* and *HWA2* loci exhibit growth termination and leaf yellowing after the seedlings developed three or four leaves. However, the plant growth phenotypes were not described in detail. In contrast, the present study determined that F_1_ hybrids from the cross of ‘A.D.T.14′ and ‘P.T.B.7′ rice exhibited limited growth and tiller number, as well as and leaf yellowing (Figure 1, Figure 2 and Figure 3). Even though leaf yellowing was also reported by Oka [4], the timing of the yellowing process was different [4]. In the present study, leaf yellowing was observed in F_1_ hybrids that had developed seven or eight leaves at 30 DAS and, furthermore, was associated with the downregulation of photosynthesis-related genes (Figure 8).

In *O. sativa*, three other gene sets have been reported to cause hybrid weakness, and the phenotypes associated with each system are different. More specifically, the hybrid weakness associated with the *HWC1* and *HWC2* loci is characterized by short stature, short roots, and rolled leaves [26], whereas that associated with the *HWI1* and *HWI2* loci is characterized by short stature and impaired root formation [2], and that associated with the *HW3* and *HW4* loci is characterized by short culms, fewer panicles, pale green leaves, and chlorotic leaf spots [3]. Remarkably, leaf yellowing has only been reported for the hybrid weakness associated with the *HWA1* and *HWA2* loci. Together, these reports suggest that either the causal genes of each system have different functions or the processes downstream of the causal gene interactions are different.

In the present study, the usefulness of SPAD value for determining the progression of leaf yellowing during hybrid weakness associated with the *HWA1* and *HWA2* loci were evaluated. SPAD values generally corresponded with leaf yellowing (Figure 4A,B) but failed to identify significant differences between the chlorophyll content of Stage-0 leaves and that of either the bases of Stage-1 leaves or the middles or bases of Stage-2 leaves. These results indicate that spectrophotometry is more sensitive than SPAD values to changes in chlorophyll content (Figure 4B,C). However, it is important to note that, because SPAD value accurately reflected degree of leaf yellowing and because spectrophotometry requires leaf destruction (Figure 4), SPAD value measurement is an effective and nondestructive method that can be used to quickly and easily evaluate hybrid weakness associated with the *HWA1* and *HWA2* loci.

The hybrid weakness phenotype that was studied by the present study also exhibited cell death in the yellow leaves (Figure 4D and Figure 5). Similarly, the hybrid weakness associated with the *HWI1* and *HWI2* loci involved cell death at the basal nodes [2], and the hybrid weakness associated with the *HW3* and *HW4* loci involved cell death in leaves [3]. Cell death has been also detected in the leaves of intraspecific *Arabidopsis* hybrids that exhibit hybrid necrosis [7,27] and in the leaves, stems, and roots of interspecific *Nicotiana* hybrids that exhibit hybrid lethality [28,29]. Therefore, despite differences in localization, cell death appears to be a common feature of hybrid weakness in plants.

In the present study, cell death was only detected in Stage-2 and Stage-3 leaves, which indicates that the timing of cell death does not coincide with that of either leaf yellowing or reductions in SPAD value or chlorophyll content (Figure 4). On the other hand, H_2_O_2_ was detected in Stage-1, -2, and -3 leaves that exhibited yellow leaf tips and low SPAD values (Figure 6). H_2_O_2_ commonly triggers plant cell death during hypersensitive reactions, senescence, abiotic stress responses, and development [30,31,32]. We detected that reactive oxygen species would lead to cell death on hybrid weakness by *HWA1* and *HWA2*.

In many cases of hybrid weakness, one of the causal genes encodes an *R* gene, and the interaction of the *R* gene with another causal gene triggers an autoimmune response [7,27,33,34]. In the hybrid weakness associated with the *HWI1* and *HWI2* loci, the causal genes include the LRR-RLK gene (*R* gene) and a subtilisin-like protease gene, respectively, and the interaction of the causal genes results in an autoimmune response [2]. Meanwhile, in the hybrid weakness associated with the *HW3* and *HW4* loci, *HW3* encodes a calmodulin-binding protein, and even though the gene is a defense-response gene, not an *R* gene, the interaction of *HW3* with *HW4* results in an autoimmune response [3]. Furthermore, in the hybrid weakness associated with the *HWA1* and *HWA2* loci, a candidate region, which harbored both loci, also contained 12 *R* genes, along with many other genes [17]. During hypersensitivity reactions, reactive oxygen species are produced, thereby mediating cell death, chloroplast disruption, and the upregulation of defense-related genes [35,36]. In the present study, cell death was detected in the F_1_ leaves after H_2_O_2_ generation and leaf yellowing (Figure 3, Figure 5, and Figure 6), the yellow leaves of the F_1_ hybrids exhibited upregulated defense-related genes (Figure 8). These results suggest that the hybrid weakness associated with the *HWA1* and *HWA2* loci involves hypersensitive reaction-like responses. However, because leaf senescence is also associated with H_2_O_2_ production, cell death, the upregulation of certain defense genes, and leaf yellowing [37], it is possible that the hybrid weakness associated with the *HWA1* and *HWA2* loci involves premature senescence. Additional molecular studies will reveal the exact mechanism underlying the hybrid weakness associated with the *HWA1* and *HWA2* loci.

## 4. Materials and Methods

### 4.1. Plant Materials and Growth Conditions

The *Oryza sativa* Indian cultivars ‘A.D.T.14′ (*indica* [17]), which is homozygous for the *Hwa1-1* allele, and ‘P.T.B.7′ (*aus* [17]), which is homozygous for the *Hwa2-1* allele, were crossed to generate F_1_ hybrid offspring. The genotypes of the two cultivars were previously reported by Oka [4]. F_1_ seeds were obtained by crossing ‘P.T.B.7′ (♀) and ‘A.D.T.14′ (♂) parents. Seeds of ‘A.D.T.14′, ‘P.T.B.7′, and the F_1_ offspring were sown on 17 July 2011. After the seeds were germinated on moistened filter paper in Petri dishes, the seedlings were transplanted to soil (Sukoyaka-Jinko-Baido; Yanmar Co., Ltd., Osaka, Japan) in Wagner pots of 1/5000 a. The seedlings were grown under natural light conditions in a greenhouse at Osaka Prefecture University, Sakai, Japan. The temperature and humidity of the greenhouse were recorded using a data logger (Ondotori; T&D Co., Ltd., Matsumoto, Japan), and the plants were fertilized weekly using Otsuka-A prescription (OAT Agrio Co., Ltd., Tokyo, Japan), which contained 18.6 mM N, 5.1 mM P, 8.6 mM K, 8.2 mM Ca, and 0.4 mM Mg. The plants were cultivated for 140 DAS to survey plant height, plant age in leaf number, tiller number, days to heading, and SPAD value. The plant height was measured from the surface of the soil to the tip of the tallest leaves. To evaluate the relationship between leaf yellowing and physiology, the leaves were classified according to degree of leaf yellowing. Leaves in which 0%, 25%, 50%, or > 75% of the blade had turned yellow were assigned to Stages 0, 1, 2, and 3, respectively (Figure 4A). These leaves classified according to degree of leaf yellowing were used to measured SPAD value and chlorophyll content, as well as to detect dead cells and H_2_O_2_. Parts of seedlings were cultivated in Wagner pots of 1/10,000 a in an incubator (14 h natural light and 10 h dark, 28 °C, light intensity: 512 µmol m^−2^ s^−1^), and at 70 DAS, these plants were used as material for gene expression analysis.

### 4.2. SPAD and Chlorophyll Measurement

A SPAD meter (SPAD-502; Konica Minolta, Inc., Tokyo, Japan) was used to measure the SPAD values of the leaves without causing damage. SPAD values were obtained from the tip, middle, and base of each leaf. Meanwhile, total chlorophyll content was measured using a previously described spectrophotometric method [38]. Briefly, the leaves were cut into small pieces, weighed, treated with 20 mL 80% acetone, and ground using a pestle until bleached. The resulting solutions were transferred to 1.5 mL tubes and centrifuged at 10,000 g for 5 min. Each supernatant was transferred to a cuvette, and the absorbance of each supernatant was measured at 663.6 and 646.6 nm, after the spectrophotometer (V-530; JASCO Corp., Hachioji, Japan) was zeroed at 750 nm. Total chlorophyll concentration (mg g^−1^ FW) was calculated using the following equation: [(17.76 × OD_646.6_ + 7.34 × OD_663.6_) × extraction volume in a cuvette]/fresh weight (g).

### 4.3. Ion leakage Measurement

Ion leakage was measured, as described previously [39]. Leaf disks (3 cm^2^) were taken from the tips, middles, and bases of the leaves, floated for 5 min in water that contained 0.2% (v/v) Tween 20 for removing ion generating on making leaf disks, transferred to Petri dishes that contained fresh water with Tween 20 (0.2%), and incubated for 3 h for leaking out ions by cell death. Their conductivity (value A) of the solutions was measured using a conductivity meter (Twin Cond B-173; Horiba, Ltd., Kyoto, Japan). The leaf disks were then incubated at 95 °C for 25 min, for leaking out ions of whole leaf disks by destroying whole organization, and cooled to room temperature, and their conductivity of the solutions was also measured (value B). Finally, ion leakage (%) was calculated using the following equation: (value A/value B) × 100%.

### 4.4. Trypan Blue Staining

Trypan blue staining was performed as described previously [40]. Detached leaves were stained by boiling for 8 min in a 1:1 (v:v) mixture of ethanol and lactophenol (i.e., alcoholic lactophenol) that contained 0.1 mg ml^−1^ trypan blue, cleared in 70% chloral hydrate solution overnight, and then preserved in 70% glycerol. Trypan blue stains dead cells. Transverse slices were prepared using a hand-section method and visualized using a light microscope (Olympus BX50; Olympus, Co. Ltd., Tokyo, Japan).

### 4.5. Detection of Hydrogen Peroxide Accumulation

Hydrogen peroxide was detected visually, using previously described methods [41]. Briefly, leaves were soaked in a 3,3-diaminobenzidine (DAB) solution for 24 h, transferred to boiling 96% ethanol until bleaching, and then visualized. The presence of H_2_O_2_ was indicated by brown staining.

### 4.6. Real-Time qRT-PCR

Total RNA was isolated from leaves using an RNAiso PLUS kit (Takara Bio, Inc., Shiga, Japan), according to the manufacturer’s protocol and then treated with RNase-free DNase (Promega Co., Madison, USA), and first-strand cDNA was synthesized from total RNA (2 µg) using oligo (dT)_18_ primers and ReverTra Ace (Toyobo Co., Ltd., Osaka, Japan). Real-time RT-PCR was carried out to analyze the expression of 11 defense-related genes and four photosynthesis-related genes (Table 1), using *Actin* as an internal control. The primers used to amplify *PR1A, PR1B, Gns5, PR4*, and *Actin* had been reported previously [42], and the other primers were designed based on RAP-DB locus ID using the Primer-BLAST design tool [43] (Table S1). Real-time RT-PCR was performed in 20 μL reaction mixtures that contained 10 μL KAPA SYBR FAST qPCR Master Mix (2×) ABI PRISM (Takara Bio), 10 µM of each forward and reverse primer (0.4 µL each), and 1 μL cDNA template, and the real-time PCR amplification was performed under the following conditions: Initial denaturation at 94 °C for 10 min, followed by 40 cycles of 15 s at 94 °C and 1 min at 60 °C, with a final 30 s extension at 72 °C using an Applied Biosystems 7300 Real-Time PCR System (Applied Biosystems, Foster, CA, USA). The results were analyzed using ABI Prism software (Applied Biosystems). Each gene expression level was divided by the expression level of *Actin* to calculate relative expression level.

### 4.7. Statistical Analysis

Data were analyzed using SPSS (version 22; IBM, Co., Armonk, USA). Tukey HSD tests were used to compare SPAD, chlorophyll content, and ion leakage values, and two-tailed Student’s t-tests were used to compare mid-parental (mean of ‘A.D.T.14′ and ‘P.T.B.7′) and hybrid values of plant height, foliar age, and tiller number.

## Figures and Tables

**Table 1:** Genes analyzed for RT-PCR.

Gene Symbol	RAP-DB locus ID ^a^	CGSNL Gene Name ^b^	Description
*PR1A*	Os07g0129200	*PATHOGENESIS-RELATED GENE 1A*	Similar to Pathogenesis-related protein PR1a
*PR1B*	Os01g0382000	*PATHOGENESIS-RELATED GENE 1B*	Similar to Pathogenesis-related protein PRB1-2 precursor
*Gns5*	Os01g0940700		Similar to Glucan endo-1,3-beta-glucosidase GII precursor
*Gns2*	Os01g0944900		Similar to Glucan endo-1,3-beta-D-glucosidase
*OsEGL2*	Os01g0942300		Similar to Beta glucanase precursor
*PR4*	Os11g0592200	*PATHOGENESIS-RELATED GENE 4*	Similar to Chitin-binding allergen Bra r 2
*CHT9*	Os05g0399400	*CHITINASE 9*	Chitinase 9
*CHT11*	Os03g0132900	*CHITINASE 11*	Similar to Chitinase 11
*RIXI*	Os11g0701800		Chitinase III C10701-rice (Class III chitinase homologue)
*ACO2*	Os09g0451000	*AMINOCYCLOPROPANE-1-CARBOXYLIC ACID OXIDASE 2*	Similar to 1-aminocyclopropane-1-carboxylase 1
*PDC1*	Os05g0469600	*PYRUVATE DECARBOXYLASE 1*	Similar to Pyruvate decarboxylase
*PSAF*	Os03g0778100	*PHOTOSYSTEM I SUBUNIT*	Similar to Photosystem-1 F subunit
*LHCB*	Os03g0592500		Similar to Photosystem II type II chlorophyll a/b binding protein
*OsRbcL*	Os06g0598500		Similar to Ribulose bisphosphate carboxylase large chain precursor
*Fd1*	Os08g0104600		Ferredoxin I, chloroplast precursor
*Actin*	Os05g0438800		Similar to Actin1

^a^ Identity of each gene was referenced using the Rice Annotation Project database (https://rapdb.dna.affrc.go.jp/); ^b^ CGSNL (Committee on Gene Symbolization, Nomenclature and Linkage, Rice Genetics Cooperative) gene names were referenced using Oryzabase (https://shigen.nig.ac.jp/rice/oryzabase/) [25].

## Supplementary Materials

The following are available online at https://www.mdpi.com/2223-7747/8/11/450/s1, Table S1: Real-time RT-PCR primers.
