# Supplementary material for: HWA1- and HWA2-Mediated Hybrid Weakness in Rice Involves Cell Death, Reactive Oxygen Species Accumulation, and Disease Resistance-Related Gene Upregulation
Source: Plants (Basel). 2019 Oct 25;8(11):450. doi: 10.3390/plants8110450 (PMC6918435; doi:10.3390/plants8110450)
Supplement: Supplementary file 1 [file plants-08-00450-s001.pdf]

Table S1. Real-time RT-PCR primers.

Gene	Forward Primer	Reverse Primer	Source/Reference
PR1A	TCGTATGCTATGCTACGTGTTT	CACTAAGCAAATACGGCTGACA	[40]
PR1B	ACGGGCGTACGTACTGGCTA	CTCGGTATGGACCGTGAAG	[40]
Gns5	AAGATTGTTCTGAGAAGAGATCGATCGA	ACTACGCGAAAATAGGTCTGGTAAACTT	[40]
Gns2	CGGCGGGCTGAGCTACACGAA	TGGGCGACGTGGTCGATCAGA	This study
OsEGL2	GGCGGTGGCGAAGCTCGGGGG	TTCTGGTTGAATGTCATGGCG	This study
PR4	GATGCCAACAAACCGTTGTC	CGCAATTATTGTCGCACCTG	[40]
CHT9	GGCGGGTTGGAGTGCGGGTTC	GCTGTTGAACGGCCTCTGGTC	This study
CHT11	TTCCCTAGATTCGGCACGACC	AAGCAAAGGGTCCATCAGGAG	This study
RIXI	GGAAGAACGACAACGTGTTCA	CAGAGCATGATTCCGCCATAG	This study
ACO2	ATGGCGGCAGCATTGTCGTTC	AGAAGCCCCAGCTCTCGCATG	This study
PDC1	TAAGGGAGAGCAAGCCCGTCT	TGGCGAGATGAATAGTGGCAC	This study
PSAF	TGCAAGGAGTCCAAGGCGTTC	CTTGCCGTAGTTCTCGAAGCG	This study
LHCB	ACGGGGTGAAGTTCGGGGAGG	GCGGTAGCCCTCGACGAATCC	This study
OsRbcL	TCTTCTACTGGTACATGCGAA	TCAACAAAACCTAAAGTCATA	This study
Fd1	ACAAGCTGGGAGACAGGCTGC	GGAGTAAGGCAGGTCGATCCC	This study
Actin	ATCCTTGTATGCTAGCGGTCGA	ATCCAACCGGAGGATAGCATG	[40]
